# Using Rasch measurement theory to assess the psychometric properties of a depressive symptoms scale in Norwegian adolescents

**DOI:** 10.1186/s12955-020-01373-5

**Published:** 2020-05-07

**Authors:** Annette Løvheim Kleppang, Anne Mari Steigen, Hanne Søberg Finbråten

**Affiliations:** 1 Department of Public Health and Sport Sciences, Faculty of Social and Health Sciences, Inland Norway University of Applied Sciences, PO Box 400, N-2418 Elverum, Norway; 2 Department of Health and Nursing Sciences, Faculty of Social and Health Sciences, Inland Norway University of Applied Sciences, PO Box 400, N-2418 Elverum, Norway

**Keywords:** Adolescents, Depression, Rasch measurement theory, Psychometrics, Surveys and questionnaires

## Abstract

**Background:**

Scales measuring depressive symptoms in adolescents and adults are widely used for epidemiological purposes. The purpose of this study is to use Rasch measurement theory to examine the psychometric properties of a six-item scale intended to measure depressive symptoms in Norwegian adolescents.

**Methods:**

The study is based on cross-sectional data from Ungdata, a survey conducted by the Norwegian Social Research Institute in cooperation with Regional Centres for Drug Rehabilitation in 2017. The target group comprised 13- to 19-years olds in Norway. Six items with four response categories, intended to measure depressive symptoms, were analysed. The analysis focused on invariance, including differential item functioning across gender and school levels. In addition, targeting, possible multidimensionality, response dependency, and the categorisation of the items were analysed.

**Results:**

The scale measuring depressive symptoms shows good reliability and, on the whole, the items work well. However, one item, ‘had sleep problems’, clearly misfit and another, ‘worried too much about things’, works differently for males and females.

**Conclusions:**

The scale has the potential to measure depressive symptoms in adolescents though there is room for improvement. To further improve the scale, the item concerning sleep problems should be rephrased.

## Background

Depression is one of the major public health challenges around the world and involves considerable economic, societal, and individual costs [1]. Worldwide, it is estimated that 10–20% of young people experience mental health problems [2]. In Norway, 16% of adolescents between the ages of 13 and 19 show depressive symptoms; this proportion has been increasing in recent years [3]. This may have serious consequences as mental health problems increase the risk of marginalisation from school and the labour market, leading to further social and economic marginalisation [4]. Adolescents with depressive symptoms also have a higher risk of mental and physical problems later in life, along with a higher risk of drug-related problems [1, 5, 6]. Several studies have found that females have a higher risk of depressive symptoms than males do [3, 7, 8].

In population-based surveys, questionnaires measuring mental health are frequently used to collect self-reported data. Measurements assessing depressive symptoms are important to identify those at risk, develop prevalence profiles, and identify where services may be required. Such knowledge is needed to achieve a knowledge base for preventing mental health problems. Good measurement scales are therefore important for both research and clinical purposes. To provide valid and reliable results to generate recommendations for practice and policies, scales with sound psychometric properties are required [9].

A wide range of scales measuring depressive symptoms exists, for example, the Beck Depression Inventory [10] and the Depressive Mood Inventory [11]. In addition, there exist several scales for measuring psychological distress in which items for measuring depressive symptoms are included, such as the Hopkins Symptom Checklist–10 (HSCL–10) [12]. The Depressive Mood Inventory shares five items with the HSCL–10. In a national survey of adolescents in Norway (Ungdata), six items derived from the Depressive Mood Inventory are used to measure depressive symptoms. However, the wording of the items differs slightly. The items are used both as single items and as a scale that provides a total score for depressive symptoms [3, 13]. Previous studies have reported that the six items measuring depressive symptoms have acceptable reliability (Cronbach’s α = 0.88) [13, 14].

The Depressive Mood Inventory has been validated using confirmatory factor analysis (CFA) in Norwegian adolescents aged 16 to 17, and was found to have acceptable fit (comparative fit index: 0.96–1; Tucker–Lewis index: 0.93–0.99; and root mean square error of approximation: 0.029–0.077). An acceptable Cronbach’s alpha was also reported: α = 0.83 [15]. However, we have not found any studies reporting psychometric properties of the Depressive Mood Inventory by means of Rasch measurement theory (RMT). RMT is a type of modern test theory named after the Danish mathematician Georg Rasch [16] and involves testing data against the probabilistic Rasch model [17, 18]. The Rasch model exists independently of the data and could form an external criterion the data could be tested against. If the data fit the Rasch model all requirements of fundamental measurement, such as specific objectivity [19] invariance [9], additivity [20, 21] and sufficiency [22], are satisfied. If data fit the Rasch model, the total score contains all the information about the person parameter and provide sufficient statistics for the persons [23]. A key feature of RMT is that person and item estimates can be measured on the same metric scale [23]. To make valid comparisons across different groups when it comes to a latent trait (in our survey: depressive symptoms), the items should work the same way across levels of person factors, such as gender, age and educational level, etc. [24, 25]. Otherwise, comparisons of scores across levels of person factors may be invalid. Such violation of the requirement of invariance is called differential item functioning (DIF) [25]. Moreover, RMT can provide detailed information at the item level [26], and seems to provide precise person estimates of the construct being measured [27].

The psychometric properties of HSCL–10 have been evaluated using RMT in Norwegian adolescents aged 15 to 16 [28], the HSCL–10 generally displayed good reliability (Cronbach’*α* of 0.85 and 0.91 for data obtained in 2001 and 2009, respectively, and Person Separation Index (PSI) of 0.59 and 0.74 in 2001 and 2009, respectively). To our knowledge, neither the Depressive Mood Inventory nor the scale measuring depressive symptoms used in Ungdata has been validated using RMT. The quality of scales measuring depressive symptoms is important if knowledge of these among adolescents is to be valid and reliable. The purpose of this study is to use RMT to examine the psychometric properties of a six-item scale intended to measure depressive symptoms in Norwegian adolescents.

## Methods

### Data collection and study population

This study is based on information retrieved from Ungdata, a survey conducted by the Norwegian Social Research (NOVA) institute in cooperation with Regional Centres for Drug Rehabilitation (KoRus). Ungdata is an annual national cross-sectional survey designed to collect data at the municipal level in Norway. The study is funded by the Norwegian Directorate of Health, the Ministry of Children, Equality, and Social Inclusion and the Ministry of Justice and Public Security. Ungdata was initiated in 2010, and, since then, has been conducted in adolescents (aged 13–19) in lower and upper secondary schools across the country [29]. The Ungdata survey covers various aspects of adolescents’ lives, such as depressive symptoms, health issues, relationships with parents and friends, leisure time activities, the local environment, and school issues. It has become an important source of information on adolescents’ well-being and health at both the municipal and national levels. Results from Ungdata are used in recommendations for policy, practice, and are frequently reported in media.

All secondary schools are invited to participate in the Ungdata survey. Participation is voluntary, and both parents and adolescents are informed in advance by means of an information letter. Since Ungdata began in 2010, 439,200 respondents have participated. In the present study, 28,105 adolescents responded to the items measuring depressive symptoms. Data collection was undertaken in lower and upper secondary schools in the eastern part of Norway during March 2017. Both rural and urban municipalities were included. The adolescents completed an anonymous web-based questionnaire at school. A teacher or an administrator was available for questions. The questionnaire was completed in Norwegian.

### Measuring depressive symptoms

Depressive symptoms were measured using six items derived from the Depressive Mood Inventory [11], which was in turn, derived from the Hopkins Symptom Checklist [12]. The adolescents were asked whether, during the previous week, they had been affected by any of the following: ‘felt that everything is a struggle (item 1)’, ‘had sleep problems (item 2)’, ‘felt unhappy, sad or depressed (item 3)’, ‘felt hopelessness about the future (item 4)’, ‘felt stiff or tense (item 5)’, and ‘worried too much about things (item 6)’. The six items have four response categories: ‘not been affected at all (1)’, ‘not been affected much (2)’, ‘been affected quite a lot (3)’, and ‘been affected a great deal (4)’. Higher scores indicate higher levels of depressive symptoms.

### Rasch measurement theory

Data were analysed against the partial credit parameterization [30] of the unidimensional polytomous Rasch model [16]. The average item-location estimate is set to 0.0 in all analyses.

The Person Separation Index (PSI) was used in this study as a reliability indicium. The PSI is analogous to Cronbach’s α, but is based on a non-linear transformation of the raw scores and could be measured despite missing values. A high PSI value indicates high reliability (consistency) and means that the scale is able to separate persons along the latent trait [24].

Rasch measurement theory includes several tests to examine the psychometric properties of the scale. Included in this study were tests of local independence (unidimensionality and response dependence), targeting, item fit, ordering of response categories, and the presence of DIF.

### Dimensionality and response dependence

To examine dimensionality of the depressive symptoms scale, the procedure of combined principal component analysis (PCA) of residuals and paired *t*-test was used. Based on the PCA, two subsets of the scale were established, and the person estimates for the two subsets were compared using paired *t*-test. If the proportion of individuals with significantly different person-location estimates on the pair of compared subscales exceeds 5%, multidimensionality in data is present [31, 32]. Residual correlations between two items > 0.3 were used as possible indicators of ‘significant’ response dependence [33]. In such cases, a response to an item is dependent on the response to another item, and might indicate that the scale is collecting redundant information. Hence, it could be considered whether one of the items should be deleted.

### Targeting

Targeting indicates how well a scale captures the person estimates. The targeting of the scale measuring depressive symptoms was assessed by comparing the distribution of the item threshold estimates to the distribution of the person estimates and was assessed both graphically and statistically*.* Mean person location values around zero indicate that the scale is well-targeted [18]. A positive value of mean person location indicates that that the sample is located at a higher level, having more depressive symptoms than captured by the average difficulty of the items. A negative value of mean person location suggests the opposite [18]. Bad targeting might bring lower reliability, and the scale might have problems to differentiate people according to their proficiency [24].

### Item fit

Item fit was examined using chi-square statistics and standardised residuals based on comparisons between observed and expected values. Items with low chi-square values and probability values higher than a Bonferroni-adjusted 5% were considered to have adequate fit with the Rasch model [18]. Item residuals between − 2.5 and + 2.5 indicate adequate item fit. In addition, item-characteristic curves (ICCs) were inspected to assess item fit*.*

As our study is based on a relatively large sample, there might be a danger of drawing false conclusions regarding item fit since significance tests such as chi-square are sensitive to sample size [34]*.* When large samples are used, even very small differences between the expected values from the Rasch model and the observed data might indicate significant misfit [9]. To avoid problems with regard to sample size, Bergh [35] recommends using a random sample approach to adjust sample size. Bergh also claims that this method is more reliable than the ‘adjust sample size’ function offered by the RUMM2030 software. Since we have a large sample size in the present study, it might be expected that sample size adjustment may be necessary. We conducted the procedure in line with Bergh [35]. Hence, 10 randomly selected samples of 540 were drawn. It is recommended to calculate sample size based on the number of items multiplied by the number of thresholds multiplied by 30 persons per threshold [36]. As the present scale has six items, four response categories, and consequently three thresholds, a sample size of 540 could be deemed adequate (6 × 3 × 30 = 540).

### Ordering of response categories

The response categories are considered to be ordered when the thresholds were significantly different and in the correct order [24].

### Differential item functioning

The criteria of invariance of measurement is a central requirement of the Rasch model and means that an instrument should work in the same way for all persons, irrespective of level of person factors, such as gender, school level, and the like.

The items were examined with respect to DIF using two-way analysis of variance (ANOVA) of standardised residuals and inspecting graphical displays (i.e., ICCs). ANOVA was used to examine whether there is a significant difference among the mean residuals for levels of available person factors [23]. Analyses of DIF were performed for the person factors gender, grade, and school level (lower vs. upper secondary school). Items showing DIF were resolved by splitting the item into e.g. gender-specific items, and the opposite person factor category was treated as a non-response. The items were resolved sequentially to distinguish real DIF from artificial DIF, starting with the item having the highest F-value [37]. Statistical significance was assumed at a Bonferroni-adjusted *p* < 0.05.

The same procedure of analyses was conducted for the whole data set, as well as the randomly selected ten subsets of the data. The psychometric properties of the scale were assessed using RUMM2030 software [38].

### Handling missing data

Missing data is easily handled in RMT as the RUMM2030 transforms raw scores into person location estimates. However, this assumes that the data are missing at random and there is adequate coverage of each of the response options, which is the case in the present study.

## Results

Our sample consists of an equal proportion of males and females. Of the 28,105 respondents, 60% were recruited in lower secondary school (aged 13–16), whereas 40% were recruited in upper secondary school (aged 16–19; Table 1).

**Table 1:** Sample characteristics (*n* = 28,105)

Characteristic	*n* (%)
Gender	
male	13,171 (47)
female	13,243 (47)
missing	1691 (6)
Education	
lower secondary school	16,746 (60)
- grade 8	5754 (20)
- grade 9	5565 (20)
- grade 10	5365 (19)
upper secondary school	11,359 (40)
- year 1	4528 (16)
- year 2	3721 (13)
- year 3	3085 (11)

The combined PCA and paired *t*-test procedure indicated that the scale could be deemed to be unidimensional (the proportion of significant *t*-tests of the difference in person–location estimates between subsets of items was 2.46%). None of the items showed evidence of response dependence (none of the pairs of items had residual correlations > 0.3), which indicates that the requirement of local independence was met. The scale was found to have acceptable reliability (PSI of 0.802), and all the items had ordered response categories.

### Targeting

**Fig. 1:**
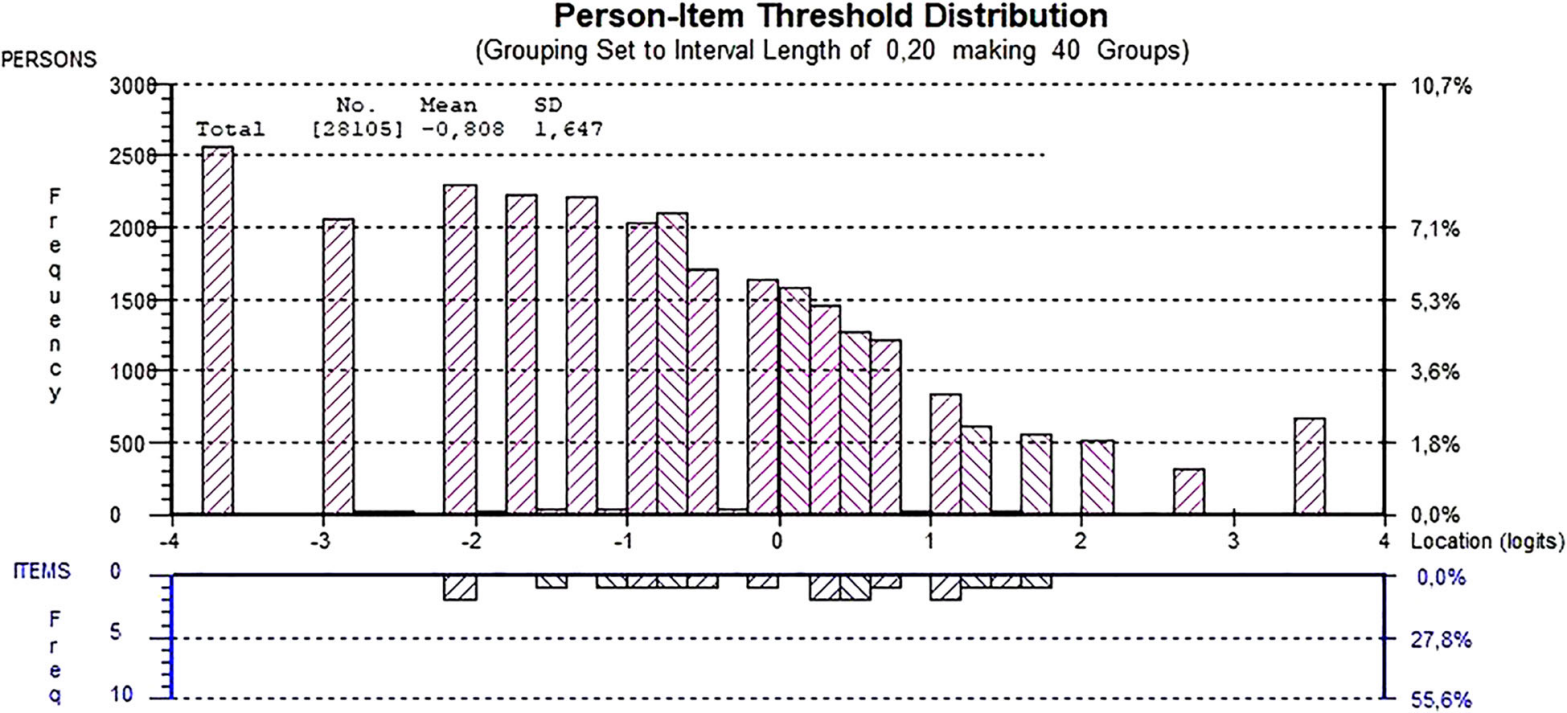
Distribution of person estimates (above the x-axis) and item threshold estimates (below the x-axis). The person estimates indicate that the adolescents (*n* = 28,105) have fewer depressive symptoms than were captured by the instrument used in Ungdata (2017)

With regard to the comparison of item and person thresholds, the item thresholds were centred around zero, while the person thresholds had a skewed distribution, with the main weight on the left (Fig. 1). The mean person location was − 0.808.

The person–item threshold distribution for lower and upper secondary schools indicated better targeting for adolescents in upper secondary school (mean value: − 0.526 logits) compared to lower secondary school (mean value: − 1.0 logits). Furthermore, the targeting was better for females (mean location: − 0.303) than for males (mean location: − 1.325).

### Item fit

According to the chi-square statistics, misfit was observed for all the items (Table 2), which was expected due to the large sample size. Inspecting location values, item 6 was the easiest to endorse, while item 5 was the hardest.

**Table 2:** Item fit statistics for the scale measuring depressive symptoms used in Ungdata

Item	Label	Location	Fit residual	*X*^2^	Probability
1	felt that everything is a struggle	−0.382	−7.856	413.027	< 0.001
2	had sleep problems	0.014	30.129	701.689	< 0.001
3	felt unhappy, sad or depressed	0.303	−11.566	524.419	< 0.001
4	felt hopelessness about the future	0.340	−7.256	292.364	< 0.001
5	felt stiff or tense	0.362	5.210	57.630	< 0.001
6	worried too much about things	−0.636	−11.282	421.640	< 0.001

**Fig. 2:**
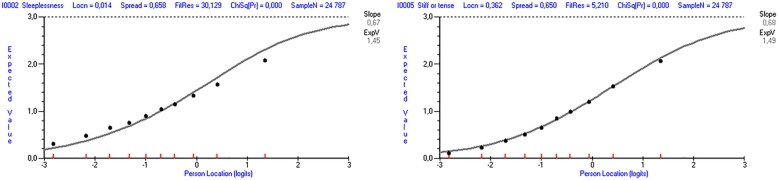
Item-characteristic curves for item 2 (left) and item 5 (right) in Ungdata 2017

According to the values of fit residuals, items 2 and 5 under-discriminated, whereas items 1, 3, 4, and 6 over-discriminated (Table 2). Comparing observed to expected values in the graphical presentation, item 5 could be deemed to have an acceptable fit, whereas item 2 under-discriminated (Fig. 2).

When 10 random samples of 540 were drawn, item 2 under-discriminated in all the samples, evidencing fit residuals of between 3.658 and 5.658. Item 5 did not under-discriminate in any of the randomly selected samples.

### Differential item functioning

**Fig. 3:**
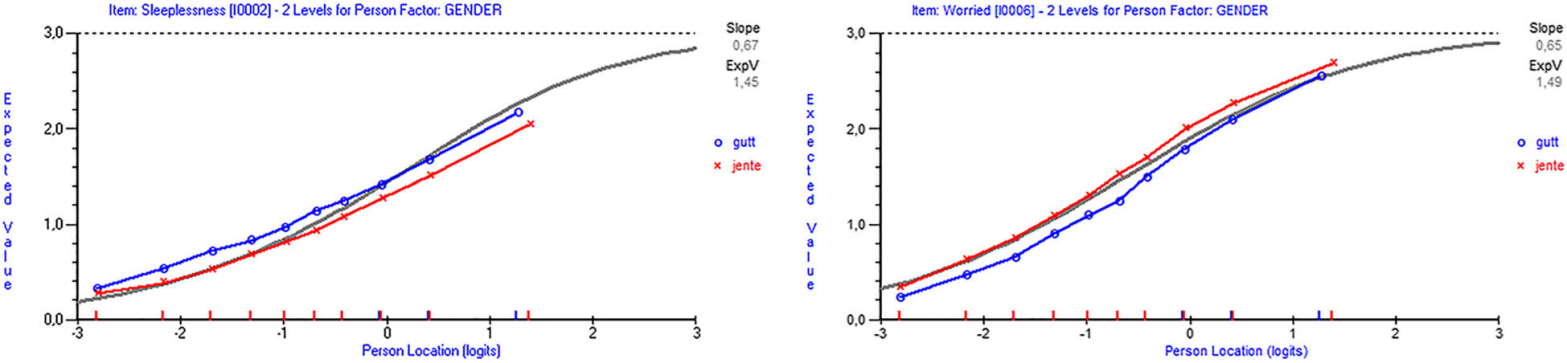
Expected value curves for item 2, and item 6 divided by gender

All items displayed DIF with regard to gender, but items 2 and 6 had very high F-values (505.05 and 739.36, respectively). In addition, all items except item 6 displayed DIF with regard to school level. Following the analysis of the randomly selected samples, only items 2 and 6 were of specific concern regarding gender DIF. Figure 3 shows the DIF for the person factor gender for items 2 and 6. Item 6 had the greatest magnitude of gender DIF and displayed DIF in all the randomly selected samples. For item 2, this was the case in seven of the ten randomly selected samples.

Males scored higher than females for item 2, whereas for item 6, females scored higher than males, given the same location of the latent trait. As the ICCs for males and females are parallel, the items show evidence of uniform DIF.

To resolve the gender DIF, item 6 was split into two separate items, one for males and one for females. The PSI was the same for both item sets (0.80). In the final step in which item 6 was resolved, the difference between the two genders is 0.09 logit smaller than the magnitude of 1.022 in the original set of six items, a change of 8.8%. After resolving the DIF for item 6, given the adjusted sample size of 540 persons, no item showed significant DIF, indicating that the DIF for item 2 was artificial.

## Discussion

At the overall level, the psychometric properties of the scale are satisfactory, and the scale has acceptable reliability (PSI). However, at a finer level, the scale shows some psychometric weaknesses related to item misfit and DIF.

### Targeting

We observed a slight mismatch between the person and item threshold distribution. However, this was expected since the scale originally is developed for clinical purposes and the present study is conducted in a healthy population. The same tendency of mismatch was observed when the HSCL–10 was validated using RMT in a general population of Norwegian adolescents [28]. However, the targeting problem is less severe for the present scale measuring depressive symptoms. Bad targeting of a scale might imply a decreased reliability index [24]. Hence, the reliability of our scale might be even better in a population where the scale is better targeted. When used for general populations of adolescents, the targeting might be improved by adding items intended to measure better and positive mental health. The targeting was also found to be better for females than for males, which is as expected as females tend to report more depressive symptoms than males [3, 7, 8]. The scale may be better adapted to females, with items that are more in line with how they might express their challenges to depressive symptoms. According to Cavanagh [39], females tend to use common verbally defined conceptions of psychological distress, whereas males tend to categorise symptoms by function and physiology.

### Item fit

All the items showed significant misfit to the Rasch model, probably due to the large sample size. After ICCs were investigated and random samples were drawn, only item 2 was of specific concern. Hence, this shows that when investigating item fit (or other analyses based on chi-square statistics), it is important to consider sample size. Item 2 under-discriminated, which is in line with Kleppang and Hagquist [28] applying RMT to the HSCL–10. Under-discriminating items are probably tapping other constructs that are negatively correlated with the latent trait [40], here depressive symptoms. There might be reasons other than depression for sleep problems, such as the use of smart devices late at night. Being on social media or gaming may also influence the answers. In addition, the item wording is very imprecise. Sleeping problems might be problems concerning falling asleep, staying asleep, waking up, or restless sleep [41]. In the course of this study, different versions of item wording were found for this item when it was used in different scales, languages, and studies [3, 11, 13, 14]. In the Depressive Mood Inventory [11] and when reporting on depressive symptoms from Ungdata in English, the wording ‘having trouble going to sleep or staying asleep’ is used [13]. However, the wording in the Norwegian version is ‘sleep problems’. Consequently, there might be problems with the translation that affect the psychometric properties of the scale. Difficulties having trouble going to sleep or staying asleep is much more precise than sleep problems, and the former item wording is preferable. For future data collection, revising this item wording is recommended, so that it is more precise and in line with the English wording of the item.

### Differential item functioning

Differential item functioning related to gender is one of the concerns revealed in this study, which is in line with Kleppang and Hagquist [28]. For items displaying DIF, further quantitative information is required and item content should be qualitatively assessed [25, 36]. There might be several reasons why item 6 (‘worried too much about things’) shows gender DIF. An explanation may be that females worry more about things than males do [42], and hence are more familiar with these kinds of problems. Alternatively, it might be easier for females than males to express their worries. On the other hand, there might be a risk of response bias as some of the adolescents might answer in the way they think is expected. The item wording may also be a source of DIF. What ‘too much’ really means can be discussed; the adolescents may have different perceptions about this. Moreover, the wording ‘worried about things’ is unclear. The adolescents may have different perceptions of what ‘things’ refers to – it may concern school, family, social relations, or health.

Splitting the item might reveal whether the DIF is real or artificial [37]. However, the consequences of creating separate response items unique to females and males, respectively, need to be considered. By splitting the item, information about depressive symptoms and the possibility of a direct comparison between males and females might be lost.

## Conclusion

The psychometric properties of the scale intended to measure depressive symptoms are satisfactory at an overall level of analysis. The scale has high reliability (PSI), the targeting is acceptable, and the response categories are ordered. However, at a finer level of analysis, the scale shows some problems related to misfit and DIF for some of the items. This study concludes that this is most likely related to the wording of the item. For future studies using the scale, we recommend that the specific items showing problems are reworded in order to strengthen the psychometric properties of the scale. Furthermore, it is recommended that psychometric analysis of this new version with reworded items is conducted. For studies for which data has already been collected, and the scale is used in its present form, this study provides valuable insights into the strengths and weaknesses of the scale – this needs to be taken into account when analysing and discussing results. It is crucial that policy and practice both in Norway and internationally are based on data from health care research that use reliable and valid scales.

## Data Availability

Availability of data and materials in the Ungdata surveys are included in a national database administered by Norwegian Social Research (NOVA). Data is available for research purposes upon application. Information on the questionnaires can also be found from the web page (in Norwegian) (http://ungdata.no/).

## Abbreviations

ANOVA: Two-way analysis of variance
CFA: Confirmatory factor analysis
DIF: Differential item functioning
HSCL-10: Hopkins Symptom Checklist–10
ICC: Item-characteristic curve
KoRus: Regional centres for drug rehabilitation
NOVA: Norwegian Social Research
PCA: Principal component analysis
PSI: Person Separation Index
RMT: Rasch measurement theory

## References

[1] Patel V, Flisher AJ, Hetrick S, McGorry P (2007). Mental health of young people: a global public-health challenge. Lancet.

[2] Kieling C, Baker-Henningham H, Belfer M (2011). Child and adolescent mental health worldwide: evidence for action. Lancet.

[3] Bakken A (2017). Ungdata 2017. Nasjonale resultater [Ungdata 2017. National results].

[4] Fergusson DM, Woodward LJ (2002). Mental health, educational, and social role outcomes of adolescents with depression. Arch Gen Psychiatry.

[5] Goodman A, Joyce R, Smith JP (2011). The long shadow cast by childhood physical and mental problems on adult life. Proc Natl Acad Sci U S A.

[6] McCarty CA, Mason WA, Kosterman R, McGorry P (2008). Adolescent school failure predicts later depression among girls. J Adolesc Health.

[7] Merikangas KR, He J-P, Burstein M (2010). Lifetime prevalence of mental disorders in US adolescents: results from the National Comorbidity Survey Replication–Adolescent Supplement (NCS-A). J Am Acad Child Adolesc Psychiatry.

[8] Wichstrøm L (1999). The emerge of gender difference in depressed mood during adolecence: the role of intensified gender socialization. Dev Psychol.

[9] Andrich D (1988). Rasch models for measurement.

[10] Beck AT, Steer RA (1984). Internal consistencies of the original and revised Beck depression inventory. J Clin Psychol.

[11] Kandel DB, Davies M (1982). Epidemiology of depressive mood in adolescents: an empirical study. Arch Gen Psychiatry.

[12] Derogatis LR, Lipman RS, Richels K, Uhlenhuth EH, Covi L (1974). The Hopkins Symptoms Checklist (HSCL) -a self-report symptom inventory. Behav Sci.

[13] Abebe DS, Frøyland LR, Bakken A (2016). Municipal-level differences in depressive symptoms among adolescents in Norway: results from the cross-national Ungdata study. Scand J Soc Med.

[14] Granrud MD, Steffenak AKM, Theander K. Gender differences in symptoms of depression among adolescents in Eastern Norway: results from a cross-sectional study. Scand J Public Health. 2017;47:157–65. 10.1177/1403494817715379 28669279

[15] von Soest T, Wichstrøm L (2014). Secular trends in depressive symptoms among Norwegian adolescents from 1992 to 2010. J Abnorm Child Psychol.

[16] Rasch G (1980). Probabilistic models for some intelligence and attainment tests. (Expanded ed.).

[17] Duncan OD, Turner CF, Martin E (1984). Rasch measurement: further examples and discussion. Surveying subjective phenomena.

[18] Tennant A, Conaghan PG (2007). The Rasch measurement model in rheumatology: what is it and why use it? When should it be applied, and what should one look for in a Rasch paper?. Arthritis Care Res.

[19] Stenner AJ (1994). Specific objectivity - local and general. Rasch Meas Trans.

[20] Perline R, Wright BD, Wainer H (1979). The Rasch model as additive conjoint measurement. Appl Psychol Meas.

[21] Andrich D, Keats JA, Taft R, Heath RA, Lovibond SH (1989). Distinctions between assumptions and requirements in measurement in the social sciences. Mathematical and theoretical systems.

[22] Andersen EB (1977). Sufficient statistics and latent trait models. Psychometrika..

[23] Andrich D, Marais I (2019). A course in Rasch measurement theory: measuring in the educational, social and health sciences.

[24] Hagquist C, Bruce M, Gustavsson JP (2009). Using the Rasch model in nursing research: an introduction and illustrative example. Int J Nurs Stud.

[25] Hagquist C (2019). Explaining differential item functioning focusing on the crucial role of external information–an example from the measurement of adolescent mental health. BMC Med Res Methodol.

[26] Tennant A, McKenna SP, Hagell P (2004). Application of Rasch analysis in the development and application of quality of life instruments. Value Health.

[27] Salzberger T, Sinkovics RR (2006). Reconsidering the problem of data equivalence in international marketing research: contrasting approaches based on CFA and the Rasch model for measurement. Int Mark Rev.

[28] Kleppang AL, Hagquist C (2016). The psychometric properties of the Hopkins Symptom Checklist-10: a Rasch analysis based on adolescent data from Norway. Fam Pract.

[29] NOVA. Ungdata. 2019 [11.02.2019]; Available from: http://www.ungdata.no/English.

[30] Masters GN (1982). A Rasch model for partial credit scoring. Psychometrika..

[31] Hagell P (2014). Testing rating scale unidimensionality using the principal component analysis (PCA)/t-test protocol with the Rasch model: the primacy of theory over statistics. Open J Stat.

[32] Smith EV (2002). Understanding Rasch measurement: detecting and evaluating the impact of multidimensionality using item fit statistics and principal component analysis of residuals. J Appl Meas.

[33] Andrich D, Humphry SM, Marais I (2012). Quantifying local, response dependence between two polytomous items using the Rasch model. Appl Psychol Meas.

[34] Lantz B (2013). The large sample size fallacy. Scand J Caring Sci.

[35] Bergh D (2015). Chi-squared test of fit and sample size-a comparison between a random sample approach and a chi-square value adjustment method. J Appl Meas.

[36] Hagquist C, Andrich D (2017). Recent advances in analysis of differential item functioning in health research using the Rasch model. Health Qual Life Outcomes.

[37] Andrich D, Hagquist C (2014). Real and artificial differential item functioning in polytomous items. Educ Psychol Meas.

[38] Andrich D, Sheridan B, Luo G (2013). RUMM2030: a windows program for the Rasch unidimensional measurement model [computer software].

[39] Cavanagh A, Caputi P, Wilson CJ, Kavanagh DJ (2016). Gender differences in self-reported depression and co-occurring anxiety and stress in a vulnerable community population. Aust Psychol.

[40] Masters GN (1988). Item discrimination: when more is worse. J Educ Meas.

[41] Gradisar M, Gardner G, Dohnt H (2011). Recent worldwide sleep patterns and problems during adolescence: a review and meta-analysis of age, region, and sleep. Sleep Med.

[42] Robichaud M, Dugas MJ, Conway M (2003). Gender differences in worry and associated cognitive-behavioral variables. J Anxiety Disord.

